# Supplementary material for: Cycling hypoxia selects for constitutive HIF stabilization
Source: Sci Rep. 2021 Mar 11;11:5777. doi: 10.1038/s41598-021-85184-8 (PMC7952589; doi:10.1038/s41598-021-85184-8)
Supplement: Supplementary file 1 — Supplementary Information [file 41598_2021_85184_MOESM1_ESM.pdf]

Cycling hypoxia selects for constitutive HIF stabilization --Supplemental Material

Mariyah Pressley[1], Jill A. Gallaher[1], Joel S. Brown[1], Michal R. Tomaszewski[2], Punit Borad[2], Mehdi Damaghi[2], Robert J. Gillies[2], Christopher J. Whelan[2*]
[1]Department of Integrated Mathematical Oncology, Moffitt Cancer Center and Research Institute, Tampa, FL 33612 USA
[2]Department of Cancer Physiology, Moffitt Cancer Center and Research Institute, Tampa, FL 33612 USA

Derivation of Eq. 5.

Solving Eq. (5) for u when the environment is normoxic (Y=1) we obtain

$$u_N(t) = u_{min} + e^{-\alpha_1 t}(u_N(0) - u_{min}) \,, \tag{S1}$$

and when the environment is hypoxic (Y=0) we obtain

$$u_H(t) = u_{max} + e^{-\alpha_0 t}(u_H(0) - u_{max}) \,. \tag{S2}$$

These two equations represent the dynamics of HIF-α levels. If the fluctuations are periodic, then the HIF-α level at the end of a period of normoxia would equal the initial HIF-α level when the environment becomes hypoxic, $u_N(T_N) = u_H(0)$, and vice versa, $u_H(T_H) = u_N(0)$. These equalities can be simplified to

$$u_N(0) = u_{max} + e^{-\alpha_0 T_H}(u_H(0) - u_{max}) \tag{S3}$$

$$u_H(0) = u_{min} + e^{-\alpha_1 T_N}(u_N(0) - u_{min}) \,. \tag{S4}$$

We can substitute Eq. (S4) into Eq. (S3) and Eq. (S3) into Eq. (S4) to solve for $u_N(0)$ and $u_H(0)$, respectively, yielding

$$u_N(0) = \frac{u_{max}(1-e^{-\alpha_0 T_H}) + u_{min}e^{-\alpha_0 T_H}(1-e^{-\alpha_1 T_N})}{1-e^{-\alpha_0 T_H - \alpha_1 T_N}} \tag{S5}$$

$$u_H(0) = \frac{u_{min}(1-e^{-\alpha_1 T_N}) + u_{max}e^{-\alpha_1 T_N}(1-e^{-\alpha_0 T_H})}{1-e^{-\alpha_0 T_H - \alpha_1 T_N}} \,. \tag{S6}$$

We can then solve for the payoff during normoxic time periods using Eq. (1) from the main text, which for normoxia simplifies to $G_N(t) = r - cu_N(t)$. Substituting in Eq. (S1) gives

$$G_N(t) = r - c[u_{min} + e^{-\alpha_1 t}(u_N(0) - u_{min})] \,. \tag{S7}$$

Then the total payoff during a normoxic period is found by integrating G_N from 0 to T_N

$$\int_0^{T_N} G_N = rT_N - cu_{min}T_N + \frac{c(1-e^{-\alpha_1 T_N})}{\alpha_1}[u_{min} - u_N(0)] \,. \tag{S8}$$

Finally, substituting Eq. (S5) in Eq. (S8) we obtain

$$\int_0^{T_N} G_N = rT_N - cu_{min}T_N + \frac{c\beta_H\beta_N(u_{min}-u_{\max})}{\alpha_1\beta}, \tag{S9}$$

which is simplified by $\beta = 1 - e^{-\alpha_0 T_H - \alpha_1 T_N}$, $\beta_N = 1 - e^{-\alpha_1 T_N}$, and $\beta_H = 1 - e^{-\alpha_0 T_H}$.

The method is the same for the hypoxic time periods, however, the payoff is slightly more complicated by the mortality term: $G_H(t) = r - cu_H(t) - \frac{m(1-q)}{k+bu_H(t)}$. Eq. (S2) can be substituted into the payoff equation to obtain

$$G_H(t) = r - c[u_{max} + e^{-\alpha_0 t}(u_H(0) - u_{max})] - \frac{m}{k+b[u_{max}+e^{-\alpha_0 t}(u_H(0)-u_{max})]}. \tag{S10}$$

Then we find the total payoff during a hypoxic interval by integrating G_H(t) from 0 to T_H, as

$$\int_0^{T_H} G_H = rT_H - cu_{max}T_H + \frac{c(1-e^{-\alpha_0 T_H})}{\alpha_0}[u_{max} - u_H(0)] + \frac{m}{\alpha_0(k+bu_{max})} ln\left|\frac{k+bu_H(0)}{e^{\alpha_0 T_H}(k+bu_{max})-b[u_{max}-u_H(0)]}\right|. \tag{S11}$$

And finally, we substitute Eq. (S6) into Eq. (S11) to obtain

$$\int_0^{T_H} G_H = rT_H - cu_{max}T_H + \frac{c\beta_H\beta_N(u_{max}-u_{min})}{\alpha_0\beta} + \frac{m}{\alpha_0(k+bu_{max})} ln\left|\frac{k\beta+b(u_{min}\beta_N+u_{max}\beta_H e^{-\alpha_1 T_N})}{k(e^{\alpha_0 T_H}-e^{-\alpha_1 T_N})+b(u_{min}\beta_N+u_{max}(e^{\alpha_0 T_H}-1))}\right|, \tag{S12}$$

again simplifing by $\beta = 1 - e^{-\alpha_0 T_H - \alpha_1 T_N}$, $\beta_N = 1 - e^{-\alpha_1 T_N}$, and $\beta_H = 1 - e^{-\alpha_0 T_H}$.

The payoff over a complete cycle is expressed as the sum of the payoff over normoxia (Eq. (S9)) and the payoff over hypoxia (Eq. (S12)), divided by total time $T = T_N + T_H$. After some rearrangement, this is simplified to

$$G = r - \frac{c}{T}(u_{min}T_N + u_{max}T_H) + \frac{c\beta_N\beta_H(u_{max}-u_{min})(\alpha_1-\alpha_0)}{\alpha_0\alpha_1\beta T} + \frac{m}{\alpha_0(k+bu_{max})T} ln\left|\frac{k\beta+b[u_{min}\beta_N+u_{max}\beta_H e^{-\alpha_1 T_N}]}{k\beta e^{\alpha_0 T_H}+b[u_{min}\beta_N+u_{max}\beta_H e^{\alpha_0 T_H}]}\right|. \tag{S13}$$

Original, unprocessed version of Figure 4A and Figure 4B:

Figure 4. Quantification of HIF-1α stabilization/de-stabilization times in vitro. Using two ovarian cancer cell lines, HIF-1α expression is found in whole lysate by Western blot analysis. Tubulin is used as control of loading the same amount of proteins. A) To measure the stabilization time, cancer cells were cultured in separate dishes, incubated in hypoxia chambers, and collected at several time points. B) To measure the destabilization time, cancer cells were grown for 72h under hypoxia, and collected at several time points after switching to normoxic conditions. See Methods for more details.

Figure 4A

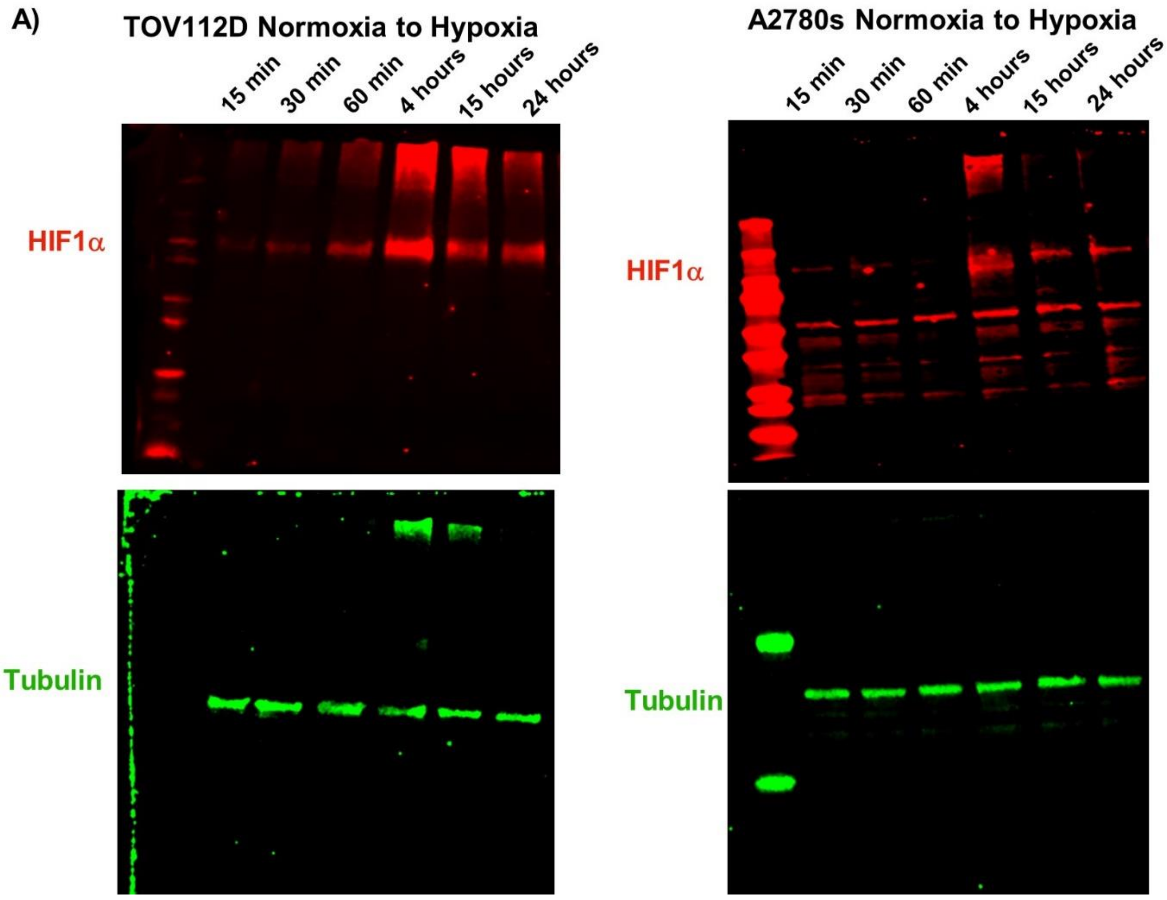

Figure 4B

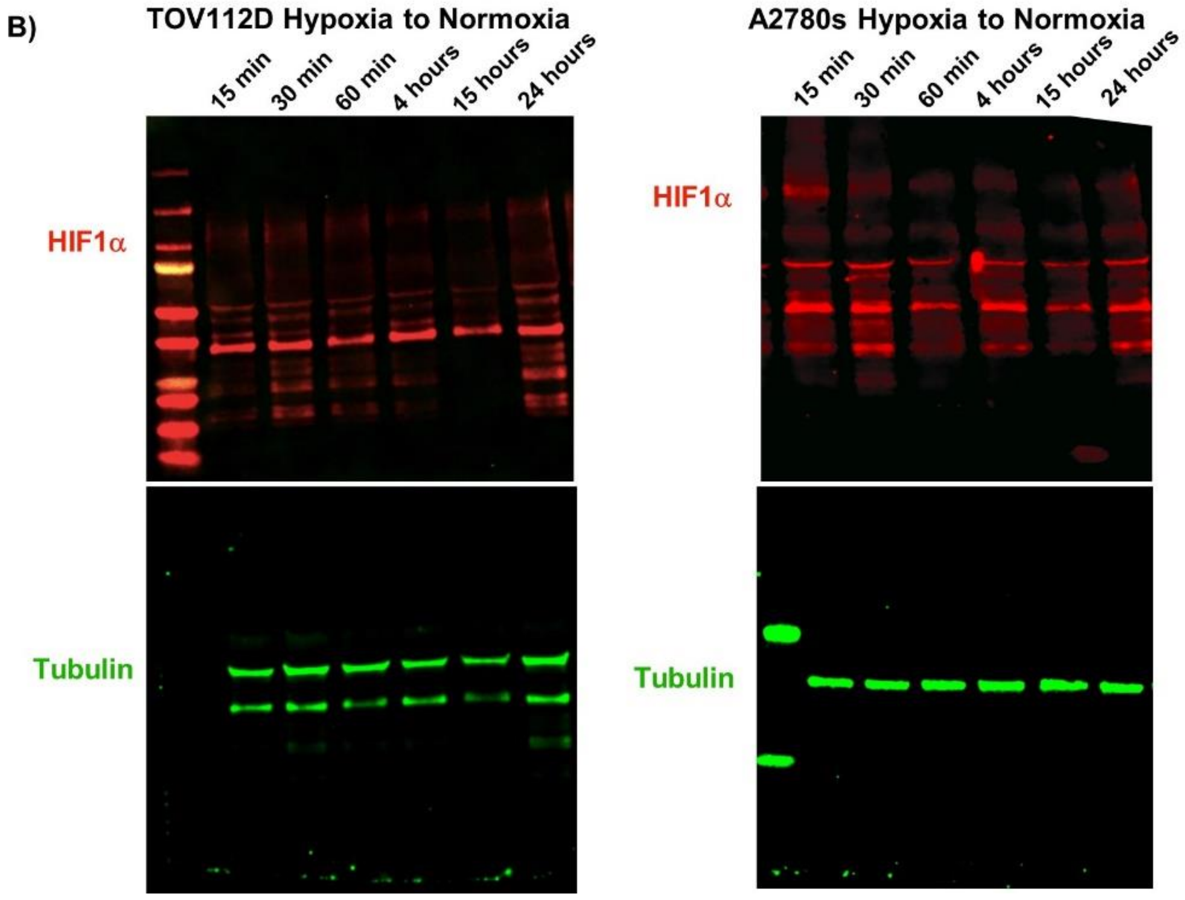
B)
TOV112D Hypoxia to Normoxia
15 min
30 min
60 min
4 hours
15 hours
24 hours
HIF1α
Tubulin
A2780s Hypoxia to Normoxia
15 min
30 min
60 min
4 hours
15 hours
24 hours
HIF1α
Tubulin

Supplemental Figure 1

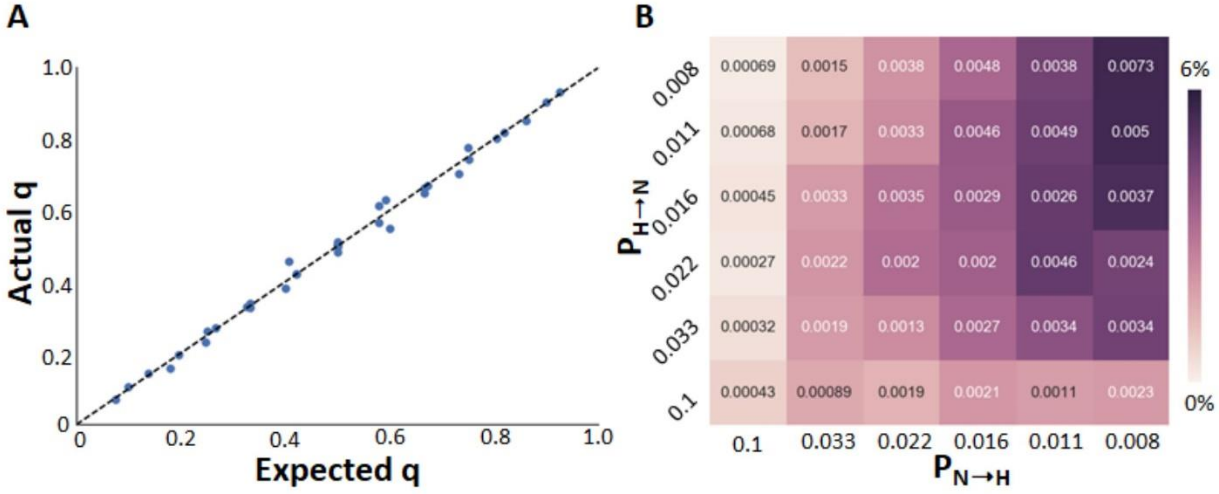

Supplemental Figure 1. The average results of 10 stochastic simulations. A) In each simulation, the probability that the environment will remain or switch its state of oxygenation is determined stochastically. We plot the expected probability that the environment will be normoxic (expected q) against the actual q, calculated after the environment is produced. B) Selective advantage of facultative HIF-α regulation in stochastic environments. Each cell is annotated with the standard error.

Supplemental Figure 2

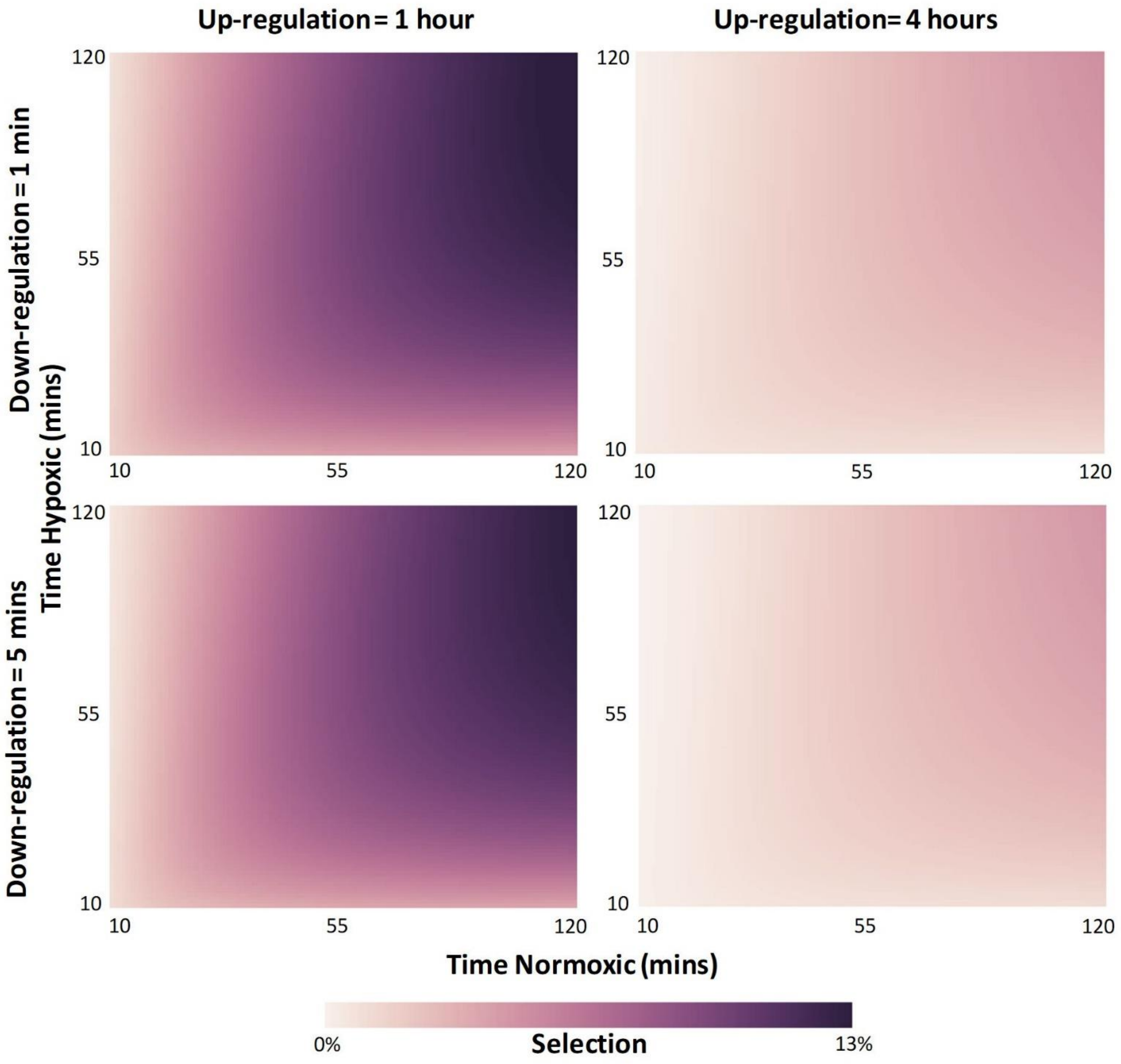

Supplemental Figure 2. Selective advantage of facultative versus constitutive HIF-α regulation in environments with fixed intervals of normoxia and hypoxia when the rates of HIF-α accumulation (α_0) and degradation (α_1) differ. For each subgraph, values of α_0 and α_1 were chosen to produce accumulation and degradation times indicated. For accumulation of 1 hour, α_0 = 0.038 min^{-1} and for accumulation of 4 hours, α_0 = 0.01 min^{-1}. For degradation of 1 min, α_1 = 2.3 min^{-1} and for degradation time of 5 min, α_1 = 0.46 min^{-1}.
